# Using AMANHI-ACT cohorts for external validation of Iowa new-born metabolic profiles based models for postnatal gestational age estimation

**DOI:** 10.7189/jogh.11.04044

**Published:** 2021-07-17

**Authors:** Sunil Sazawal, Kelli K Ryckman, Harshita Mittal, Rasheda Khanam, Imran Nisar, Elizabeth Jasper, Sayedur Rahman, Usma Mehmood, Sayan Das, Bruce Bedell, Nabidul Haque Chowdhury, Amina Barkat, Arup Dutta, Saikat Deb, Salahuddin Ahmed, Farah Khalid, Rubhana Raqib, Muhammad Ilyas, Ambreen Nizar, Said Mohammed Ali, Alexander Manu, Sachiyo Yoshida, Abdullah H Baqui, Fyezah Jehan, Usha Dhingra, Rajiv Bahl

**Affiliations:** 1 Center for Public Health Kinetics, Global Division, New Delhi, India; 2 Public Health Laboratory-IDC, Chake Chake, Pemba,Tanzania; 3 University of Iowa, College of Public Health, Department of Epidemiology, Iowa City, Iowa, USA; 4 Department of International Health, Johns Hopkins Bloomberg School of Public Health, Baltimore, Maryland, USA; 5 Aga Khan University, Department of Paediatrics and Child Health, Karachi, Sindh, Pakistan; 6 Projahnmo Research Foundation, Dhaka, Bangladesh; 7 International Center for Diarrheal Disease Research, Bangladesh, Mohakhali, Dhaka, Bangladesh; 8 World Health Organization (MCA/MRD), Geneva, Switzerland

## Abstract

**Background:**

Globally, 15 million infants are born preterm and another 23.2 million infants are born small for gestational age (SGA). Determining burden of preterm and SGA births, is essential for effective planning, modification of health policies and targeting interventions for reducing these outcomes for which accurate estimation of gestational age (GA) is crucial. Early pregnancy ultrasound measurements, last menstrual period and post-natal neonatal examinations have proven to be not feasible or inaccurate. Proposed algorithms for GA estimation in western populations, based on routine new-born screening, though promising, lack validation in developing country settings. We evaluated the hypothesis that models developed in USA, also predicted GA in cohorts of South Asia (575) and Sub-Saharan Africa (736) with same precision.

**Methods:**

Dried heel prick blood spots collected 24-72 hours after birth from 1311 new-borns, were analysed for standard metabolic screen. Regression algorithm based, GA estimates were computed from metabolic data and compared to first trimester ultrasound validated, GA estimates (gold standard).

**Results:**

Overall Algorithm (metabolites + birthweight) estimated GA to within an average deviation of 1.5 weeks. The estimated GA was within the gold standard estimate by 1 and 2 weeks for 70.5% and 90.1% new-borns respectively. Inclusion of birthweight in the metabolites model improved discriminatory ability of this method, and showed promise in identifying preterm births. Receiver operating characteristic (ROC) curve analysis estimated an area under curve of 0.86 (conservative bootstrap 95% confidence interval (CI) = 0.83 to 0.89); *P* < 0.001) and Youden Index of 0.58 (95% CI = 0.51 to 0.64) with a corresponding sensitivity of 80.7% and specificity of 77.6%.

**Conclusion:**

Metabolic gestational age dating offers a novel means for accurate population-level gestational age estimates in LMIC settings and help preterm birth surveillance initiatives. Further research should focus on use of machine learning and newer analytic methods broader than conventional metabolic screen analytes, enabling incorporation of region-specific analytes and cord blood metabolic profiles models predicting gestational age accurately.

Globally, 15 million infants are born preterm and approximately 1 million children die each year due to complications of preterm birth; of which 81% belong to low resource settings in Asia and Africa [1-3]. About 23.3 million infants (19.3% of live births) are estimated to be born small for gestational age (SGA) in Low and Middle-Income Countries (LMIC). Even reducing this to 10.0% would reduce neonatal deaths by 9.2% (254 600 deaths) [4]. In LMIC settings, estimates of gestational age (GA) at birth is central to population estimates for preterm and SGA births which in turn is critical to planning, policy, targeting interventions to reduce these outcomes, evaluation of intervention impact as well as focused care for these high-risk births [5,6].

Early pregnancy ultrasound measurements are considered to be the gold standard in determining GA, as ultrasound measurements during mid and late pregnancy are unreliable [7]. Obtaining an early accurate GA in low resource settings is challenging due to absence of ultrasound equipment and shortage of trained technicians [8]. In these settings, last menstrual period (LMP) is often used for assessment of GA. Although LMP in high income countries has an error of only few days, it has now been shown to be highly unreliable in LMIC. Low prevalence of early antenatal care and hence recall problems, high rates of conception during lactation amenorrhea and conception immediately following long duration contraception patches contributes to this inaccuracy [9]. Postnatal methods such as Dubowitz or Ballard assessment scales, used to estimate GA based on physical & neuromuscular characteristics, require trained staff and even in trained hands have low reliability & high interrater variability, particularly among SGA and preterm infants [10-13].

To address this felt need, recent studies have tried to use routine new-born screening metabolic markers for estimation of GA in developed country settings based on retrospective health services cohort analysis [6,14,15]. Ryckman et al. [6] developed a regression model with 88 parameters from metabolite screening data of 230 013 new-borns in Iowa, USA. The developed model could predict GA within 1 and 2 weeks of gestation for 78% and 95% of new-borns respectively. Wilson et al., [15] on metabolite data of 249 700 new-borns in Ontario, Canada found that the developed model including gender and birthweight as covariates, along with metabolites was able to predict GA within about 1 and 2 weeks of gestation in 66.8% and 94.9% new-borns. A similar study among 720 503 infants from California, USA was able to correctly classify 78.3% and 91.7% with 1 and 2 weeks correctly using discriminant analysis [14].

Limited evidence is currently available for validating these algorithms developed for populations in developed countries across different ethnicities [16] and in low resource settings [17]. Only one study currently exists for validation in a low resource setting, using the model developed in a Canadian population (Ontario Model) [15] which has been used for 487 new-borns in a prospective cohort from Bangladesh. The model in this study provided Root Mean Square Error (RMSE) of 1.35 weeks. An additional model which included expanded clinical data with limited feasibility (ie, new-born haemoglobin peak percentages) provided RMSE of 1.07 [17]. Evaluating these models in LMIC settings where their use is actually of importance to global health is a priority.

Alliance for Maternal and New-born Health Improvement – All Children Thrive (AMANHI-ACT) cohorts (in 3 LMIC – Tanzania, Pakistan and Bangladesh) provided a unique opportunity for evaluating external validity for this approach. In the present study, we report evaluation of the hypothesis that regression models developed at Iowa, USA [6] applied to prospective cohorts in South Asia and Sub-Saharan Africa provide valid estimation of postnatal GA and discrimination for preterm births.

## METHODS

### Study population

This validation study was conducted nested within a prospective pregnancy cohort study entitled The Alliance for Maternal and New-born Health Improvement (AMANHI) Bio-repository study. This study was carried out in three LMIC with harmonised methods/protocols to capture data on the biological determinants of adverse pregnancy outcomes and biological specimens were bio-banked for future analysis. Detailed methods of the study have been reported previously [18-20]. Briefly, trained field workers identified pregnant women by active surveillance in the field and they were enrolled in the study before 19 weeks of gestation which was confirmed by ultrasonography using the fetal crown rump length (if <14 weeks gestation) [21-24] or biparietal diameter and femur length (if ≥14 weeks) [24,25]. All enrolled women were followed prospectively through pregnancy till 45-60 days post-partum. Clinical information and bio-specimens were collected at all the time points. Birth weight was measured within 1 hour using a standard new-born weighing scale (SECA Corporation, Columbia, MD, USA).

**Figure 1:**
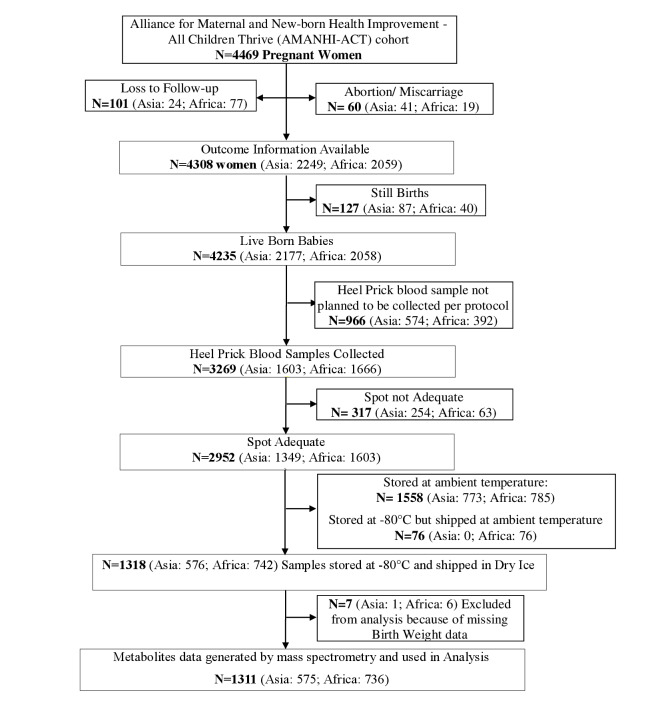
Consort cohort flow diagram for pregnancies contributing samples for this nested sub-study.

The All Children Thrive (ACT) extension of the AMANHI cohorts added follow up of new-borns until 3 years of age. As part of this change, a heel prick sample collection from neonate between 24 to 72 hours after birth was introduced to the protocol. Details of the cohort flow are provided in **Figure 1**. Based on the quality control pilot analysis, only samples from 1318 new-borns that had been stored in -80°C and were shipped in dry ice were considered for metabolic analyte estimation and processed at the collaborating laboratory in Iowa. 7 samples were excluded due to missing birthweight data.

### Ethical approval and Informed consent

The AMANHI and AMANHI-ACT study was approved by Ethics Review Committee of World Health Organization (WHO, RPC 532). These studies were also approved by local Institutional Review Boards as per regulations in each of the countries. Additional Material Transfer Agreement (MTA) requirements were approved by the appropriate committees and/or Ministry of Health as required by local regulations. A core set of written consents were obtained from pregnant women, in their local or preferred languages, for undergoing a screening ultrasound examination and subsequent enrolment if eligibility was confirmed (pregnancies between 8–19 weeks’ gestation). This included a separate consent for biological specimens, their bio-banking and subsequent use. In addition to the consent at enrolment, an additional written consent was also obtained from mothers prior to collection of heel prick samples of their new-borns.

### Sample collection and processing

Collection of blood sample was performed by a team of health care providers via heel prick method within first 24-72 hours of birth of infants. For this analysis, blood samples were spotted onto Whatman 903 protein saver cards (using a standard operating procedure, SOP), air-dried, stored at -80°C in zip-locks in freezer and shipped to University of Iowa on dry ice (with silica gel sachets in gas impermeable zipper bags). Samples were processed for metabolites analysis at State Hygienic Laboratory, Ankeny, Iowa, USA using tandem mass spectrometry for new-born metabolic screening. Forty-four metabolites including acylcarnitines, enzymes and hormones were estimated as per standard procedures used for the Iowa study and available in public domain (**Table 1**) [6]. The Laboratory and Analysis unit at Iowa was blinded to the gold standard GA. Statistical modelling analysis of the metabolite data along with available meta data was performed at University of Iowa, College of Public Health, Department of Epidemiology using the previously established model equation [6]. After the initial GA estimation, the data set was sent to the WHO coordinating centre, where the data set was linked with ultrasound based GA. The sample size had to be adjusted to feasibility as data from these settings were not available for Root Mean Square Error (RMSE) estimations, we finally based our sample size on feasibility and power of 90% to provide 5% precision in ROC analysis by region (Asia and Africa).

**Table 1:** Metabolites, their squared and cubed terms included in the metabolites model for prediction of gestational age

Amino acids	Alanine, Arginine, Isoleucine + Leucine, Methionine, Phenylalanine, Tyrosine, Valine
**Acylcarnitines**	Acetylcarnitine (C2), Propionylcarnitine (C3), Malonylcarnitine (C3-DC), Butyrylcarnitine +Isobutyrylcarnitine (C4), Methylmalonylcarnitine (C4-DC), Isovalerylcarnitine + Methylbutyrylcarnitine (C5), Tiglylcarnitine (C5:1), 3-Hydroxyisovalerylcarnitine (C5-OH), Glutarylcarnitine (C5-DC), Hexanoylcarnitine (C6), Methylglutarylcarnitine (C6-DC), Octanoylcarnitine (C8), Octenoylcarnitine (C8:1), Decanoylcarnitine (C10), Decenoylcarnitine (C10:1), Dodecanoylcarnitine (C12), Dodecenoylcarnitine (C12:1), Tetradecanoylcarnitine (C14), 3-Hydroxytetradecanoylcarnitine (C14-OH), Palmitoylcarnitine (C16), Palmitoleylcarnitine (C16:1), 3-Hydroxypalmitoylcarnitine (C16-OH), 3-Hydroxypalmitoleylcarnitine (C16:1-OH), Stearoylcarnitine (C18), Oleoylcarnitine (C18:1), 3-Hydroxyoleoylcarnitine (C18:1OH), Linoleoylcarnitine (C18:2)
**Enzymes & Hormones**	Galactose-1 Phosphate Uridyl Transferase (GALT), 17-Hydroxyprogesterone (17 OHP), Thyroid Stimulating Hormone (TSH)
**Squared Values**	Alanine, Arginine, Isoleucine + Leucine, Methionine, Phenylalanine, Valine, C2, C5, C4-DC, C5-DC, C6, C8, C8:1, C10, C12, C12:1, C6-DC, C14, C16, C16:1, C18, C18:1, C18:2, C14-OH, C16-OH, C16:1-OH, GALT, TSH, 17 OHP
**Cubic Values**	Alanine, Isoleucine + Leucine, Methionine, Phenylalanine, Valine, C2, C5, C4-DC, C5-DC, C8, C8:1, C10, C12, C12:1, C16, C16:1, C18, C18:1, C18:2, C16-OH, TSH

### Statistical analysis

Preterm births were defined as all births that occurred at <37 weeks’ gestation. SGA was defined as cases where birthweight was below the 10th percentile of the reference birthweight (within gender specific GA at delivery strata) using new intergrowth standards [26]. Low Birth Weight was defined as an infant weighing below <2500 g. Details of statistical analysis and development of the regression equations and coefficients in those equations has been already published [6]. In generation of the estimated GA, the metabolite and metadata obtained from these samples were plugged into previous equations of predictive models (using the regression coefficients derived from parent Iowa data) [6]. No imputation of data was performed for missing data and the laboratory group was blinded to the GA of the samples, which was only added to data set at WHO after the results were shared to avoid bias.

We compared estimated GA and preterm births based on output from our models using analysis of blood spots against first trimester ultrasound GA, which are considered the gold standard for GA measurement [27,28]. RMSE were estimated for the total cohort as well as Sub Saharan African and South Asian cohorts. Again, performance among SGA new-borns was separately evaluated to ascertain any effect of higher Intra Uterine Growth Restriction (IUGR) prevalent in LMIC on the model performance. Concordance/discordance by week to help comparison with other published studies was undertaken. Two models were evaluated:

**Model 1 – Metabolites model:** The final new born metabolic linear regression model consisted of 88 parameters published previously [6]. This was derived by Ryckman et al [6] initially by performing a univariate analysis for each metabolite and GA. Ryckman et al., [6] used the squared and cubic terms of each metabolite to address nonlinearity between the metabolites and the GA. The linear, squared and cubic terms of metabolites which were significant at *P* < 0.01 from the univariate analysis were used in the model-building data set. Terms with significance less than 0.05 were retained for subsequent modelling. Cubic significant terms were used only when the squared terms were significant.

**Model 2 – Metabolites and birth weight model:** In the second model, birthweight was introduced into the metabolites model to assess improved prediction over the metabolites model only.

### Receiver operating characteristic (ROC) area under curve analysis

For ROC analysis, we used Stata 16.1 (Stata Corp LLC, Texas, TX, USA) and Medcalc (MedCalc Software Ltd, Ostend, Belgium). Generation of ROC curve and Area Under the ROC Curve (AUC) estimation was performed and interpreted using standard methods [29-31]. We estimated Youden index J [32] defined as J = max (sensitivity_c_ + specificity_c_ – 1) where c ranges over all possible criterion values. Graphically, J is the maximum vertical distance between the ROC curve and the diagonal line. For both the Youden index and its corresponding criterion value, 95% confidence intervals (CI) were estimated using bootstrap methods [33-36]. Corresponding values and 95% CI for sensitivities and specificities were also estimated for a range of fixed and pre-specified sensitivities/specificities [37]. Confidence intervals were bootstrapped for 95% confidence intervals [32-35]. Comparison of ROC curves estimating difference, CI and *P*-value was also performed using bootstrap methods [38,39]. For the bootstrap estimation, we used 2000 replications and a fixed seed of 20 to enable replication of data.

## RESULTS

### Cohort characteristics of infants included in the study

A total of 1311 samples were used from the AMANHI/ACT cohort for the current analysis. As at that point in time, 3 cohorts had variable number of births left to enrol; a prior decision was made to have contribution from Bangladesh and Pakistan combined enabling analysis by region. The proportion of preterm infants was higher in South Asia as compared to Sub Saharan Africa. The proportion of infants with birthweight <2500 g was 5 times higher in South Asia as compared to Sub Saharan Africa. Characteristics of the cohort contributing to this analysis is provided in **Table 2**.

**Table 2:** Cohort characteristics of infants included in the metabolic screening study

Characteristics	All sites combined (total cohort)	Sub Saharan Africa (Tanzania)	South Asia (Pakistan & Bangladesh)
	**n = 1311**	**n = 736 (56.1%)**	**n = 575** **(43.9%)**
**Sex, n (%):**			
Male	620 (47.3)	354 (51.9)	266 (53.7)
Female	691 (52.7)	382 (48.1)	309 (46.3)
Gestational age (weeks), overall mean ± SD	38.5 ± 1.7	38.7 ± 1.7	38.4 ± 1.7
**Gestational age category (weeks), n (%):**			
Term (≥ 37 weeks)	1161 (88.6)	669 (90.9)	492 (85.6)
Preterm (< 37 weeks)	150 (11.4)	67 (9.1)	83 (14.4)
Late preterm (34 to < 37 weeks)	123 (9.4)	52 (7.1)	71(12.3)
Early preterm (**<** 34 weeks)	27 (2.0)	15(2)	12 (2.1)
Birth weight (g), mean ± SD	3053 ± 561	3267 ± 510	2778 ± 502
**Birth weight category, n (%):**			
≥2500 g	1127 (86.0)	699 (95.0)	428 (74.4)
<2500 g (low birthweight)	184 (14.0)	37 (5.0)	147 (25.6)
Twin or triplet, n (%)	36 (2.8%)	27 (3.7%)	9 (1.6%)
Age at newborn sample collection (h), mean ± SD	49.0 ± 16.2	46.6 ± 12.7	52.1 ± 19.4

SD – standard deviation, n – number, g – grams, h – hours

### Overall performance of gestational age estimation models

Model including only metabolites estimated GA relative to ultrasound-validated GA with RMSE of 1.65 weeks, model performance improved by including birth weight into it resulting in a RMSE of 1.52 weeks (**Table 3**). Regional validity of the metabolites only model was substantially lower in Africa (RMSE 1.78) compared to Asia (RMSE 1.51) while addition of birth weight to the model stabilized it across region RMSE 1.53 (Africa), 1.52 (Asia).

**Table 3:** Differences between ultrasound based and predicted gestational ages among overall and SGA infants

Performance measure	Metabolites model			Metabolites & birthweight model		
	**Total**	**Sub Saharan Africa**	**South Asia**	**Total**	**Sub Saharan Africa**	**South Asia**
**Overall:**						
RMSE	**1.65**	**1.78**	**1.51**	**1.52**	**1.53**	**1.52**
Weeks discrepant						
1 week (%)	68.7	64.7	73.9	70.5	71.7	68.9
2 weeks (%)	88.6	86.6	91.3	90.1	88.9	91.7
**SGA:**						
RMSE	**1.77**	**2.43**	**1.53**	**2.14**	**2.55**	**1.98**
Weeks discrepant						
1 week (%)	65.7	46.8	70.6	43.1	38.1	39.8
2 weeks (%)	90	79	92.4	76.3	58.8	82.8
**Estimates from previous country studies:**						
	**Ontario Canada**	**Iowa newborn screening**	**California Newborn screening**	**Bangladesh Ontario model**	**Bangladesh extended Ontario model 3**	
N	249 700	230 013	729 503	487	487	
Weeks discrepant						
1 week	66.8	78	78.3	57.3	63.9	
2 weeks	94.9	95	91.7	88.5	94.3	

Overall, inclusion of birthweight in the metabolites model (Model 2) increased accuracy of predicting GA correctly within 1 week to 70.5% and within 2 weeks to 90.1% of new-borns when compared to ultrasound based GA (**Table 3**). In terms of external validity, these estimates were reasonably valid though marginally lower than observed in original Iowa sample. The estimates were higher than those observed in Bangladesh samples where GA was estimated using the Ontario Model [15] without clinical parameters like new-born haemoglobin peak percentages, and similar to those (63.9% and 94.3%) obtained even after additional clinical parameters (**Table 3**). Amongst SGA infants, the models were less accurate overall. However, one without birth weight performed consistently better, RMSE 1.77 (Model 1) vs, 2.14 (Model 2). Similar differences were observed by region ie, Africa and Asia (**Table 3**). In general, all models predicted GAs close to full term with the highest accuracy, while tending to overestimate GA in pre-term infants and underestimate GA in post-term infants (**Table 4** and **Table 5**). The prevalence estimates of infants less than 37 weeks GA were least affected (estimated: gold standard) 10.9%: 11.4% in comparison with GA 39-40 weeks 35%: 49.8% and GA>40 weeks 0.2%: 8.2% (**Table 4**). **A **detailed week wise improvement in prediction of gestational age was also observed for the two models (**Table 6**).

**Table 4:** Prevalence of gestational age groups amongst cohorts

Weeks	Ultrasound based GA		Metabolites and birthweight model	
	N	%	N	%
**Total:**				
≤34	27	2	27	2.1
35-36	123	9.4	116	8.8
37-38	400	30.5	707	53.9
39-40	653	49.8	458	35
>40	108	8.2	3	0.2
**Sub Saharan Africa:**				
≤34	15	2	8	1.1
35-36	52	7.1	28	3.8
37-38	216	29.4	354	48.1
39-40	376	51	343	46.6
>40	77	10.5	3	0.4
**South Asia:**				
≤34	12	2.1	19	3.3
35-36	71	12.3	88	15.3
37-38	184	32	353	61.4
39-40	277	48.2	115	20
>40	31	5.4	0	0

GA – gestational age

**Table 5:** Cross tabulation (concordance) between ultrasound based and predicted gestational ages (metabolites and birthweight model only)

Ultrasound based GA (weeks)	Predicted GA (in weeks)					
	**≤34**	**35-36**	**37-38**	**39-40**	**>40**	**Total**
**Overall**						
≤34	**13 (48.1%)**	13	1	-	-	27
35-36	14	**42 (34.1%)**	61	6	-	123
37-38	-	43	**270 (67.50%)**	87	-	400
39-40	-	15	328	**307 (47%)**	3	653
>40	-	3	47	58		108
**Sub Saharan Africa:**						
≤34	**5 (33.3%)**	9	1	-	-	15
35-36	3	**4 (7.7%)**	40	5	-	52
37-38	-	7	**136 (63%)**	73	-	216
39-40	-	6	147	**220 (58.5%)**	3	376
>40	-	2	30	45	**-**	77
**South Asia:**						
≤34	**8 (66%)**	4	-	-	-	12
35-36	11	**38 (53.5%)**	21	1	-	71
37-38	-	36	**134 (72.8%)**	14	-	184
39-40	-	9	181	**87 (31.4%)**	-	277
>40	-	1	17	13	**-**	31

GA – gestational age

**Table 6:** Overall difference between ultrasound based and predicted gestational ages

Weeks Discrepant	Metabolites model			Metabolites and birth weight model		
	**N**	**%**	**Cumulative %**	**N**	**%**	**Cumulative %**
**Total:**						
0	333	25.4	25.4	352	26.9	26.9
1	568	43.3	68.7	572	43.6	70.5
2	261	19.9	88.6	257	19.6	90.1
3	94	7.2	95.8	103	7.9	98
≥4	55	4.2	100	27	2	100
**Sub Saharan Africa:**						
0	169	23	23	209	28.4	28.4
1	307	41.7	64.7	319	43.3	71.7
2	161	21.9	86.6	126	17.1	88.9
3	59	8	94.6	67	9.1	98
≥4	40	5.4	100	15	2	100
**South Asia:**						
0	164	28.5	28.5	143	24.9	24.9
1	261	45.4	73.9	253	44	68.9
2	100	17.4	91.3	131	22.8	91.7
3	35	6	97.4	36	6.3	97.9
≥4	15	2.6	100	12	2	100

**Figure 2:**
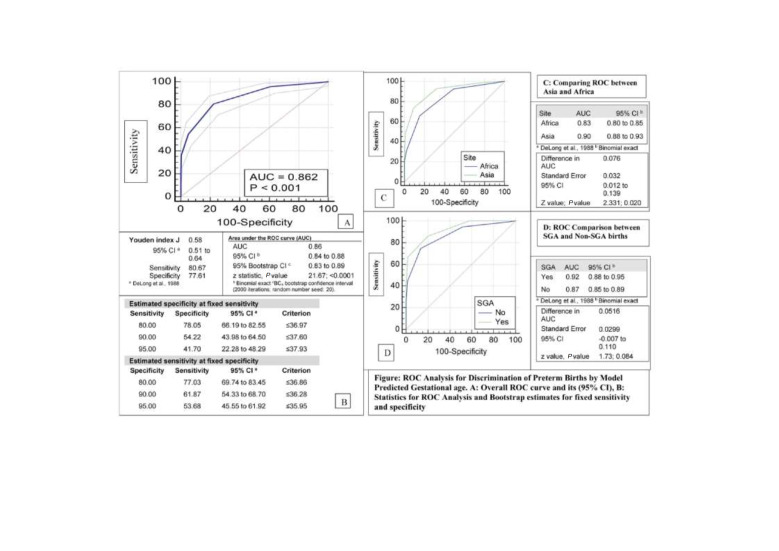
Receiver operating characteristic (ROC) curve analysis and statistics for the final regression model in discriminating pre-term births. **Panel A.** Overall ROC curve and its (95% CI) **Panel B.** Statistics for ROC analysis and Bootstrap estimates for fixed specificity and sensitivity. **Panel C.** Comparing ROC between Asia and Africa. **Panel D.** ROC comparison between small for gestational age (SGA) and non SGA births.

To evaluate the discriminatory ability of the estimated GA in identifying pre term births, ROC analysis performed indicated a highly significant ability, with area under curve of 0.86 (conservative bootstrap 95% CI = 0.83 to 0.89); *P* < 0.001) and a Youden Index of 0.58 (95% CI = 0.51 to 0.64) with a corresponding sensitivity of 80.7% and specificity of 77.6% (**Figure 2**, Panels A and B). These values were similar to those observed in Iowa study (AUC = 0.89, 95% CI = 0.89 to 0.90)] [6]. There was a small but statistically significant difference between the curves in Africa and Asia of 8% (95% CI = 1% to 14%; *P* = 0.02) (**Figure 2**, Panel C). Curves were not statistically different (within the limitation of restricted power and wide CI) between SGA and non-SGA births difference of 5% (95% CI = -1% to 11%; *P* = 0.08) (**Figure 2**, Panel D).

## DISCUSSION

This validation study affirms that the “Iowa new-born screening data based” regression modelling approach and algorithms for estimating GA [6] was effective with an accuracy of approximately 1 to 2 weeks, of ultrasound-validated GA, among infants from cohorts in East Africa and South Asia. Study observed marginally lower accuracy overall as compared to Iowa results, but a range of accuracy was consistent and marginally better than hitherto reported studies with metabolic screening approach. A more recent refinement of adding clinical indicators, including ratio of foetal to adult haemoglobin, has been reported to improve RMSE to 1.07 in Ontario model [15,16] which was not evaluated in this study. However, in context of LMIC, its inclusion may impact feasibility and cost, therefore posing a concern for external validity.

Addition of birth weight to the metabolites model improved prediction both for Africa and Asia. However, improvement was substantial in estimation for African sample as compared to Asian sample. Accuracy among SGA new-borns was much lower with overall RMSE of 2.14, more so in Africa RMSE 2.55 and it was also adversely affected by addition of birth weight to the model changing from 1.77 to 2.14. This finding is suggestive of either growth faltering affecting the metabolite levels and/or the SGA population structure of the Iowa data imposing a limitation on model generation.

Our study provides evidence that in the absence of adequate infrastructure in low resource settings, storage and transportation of samples to a reference laboratory for evaluation provides a valid option and approach. However, this approach with requirement of ultracold chain, shipment and processing costs estimated at 50.00 USD [15] does highlight feasibility restrictions.

Our study had a number of strengths and also some limitations which need consideration for interpreting the results. The strengths of this study include I) Use of samples from 3 countries representing both South Asia and East Africa, regions which are major global contributors to global mortality associated with preterm and SGA births. This being the only reported study from Africa. II) The study design was nested in a well-described population-based cohorts of pregnancy representative of the larger populations, using harmonized protocols and SOP coordinated by WHO. III) Gold standard GA assessments were extremely ideal, populations were under 2-month surveillance for early pregnancy identification with added measures like maintaining menstrual calendar (Bangladesh) and providing pregnancy test (Pemba), gold standard ultrasound examination was harmonized and undertaken early in pregnancy between 8 and 19 weeks of gestation. IV) Establishment of standard SOP for sample collection, storage and shipment based on pilot QC, simultaneously adopted and implemented by all three sites, resulting in high quality of samples received for analysis and V) Unlike other LMIC study [17], we did not perform any imputation (predictive mean matching) or winsorization (Tukey Fence approach), in our data analysis which would in fact make analysis conservative and easily reproducible in other settings. As for weaknesses I) The primary limitation of this study is the participation bias against early preterm and early deaths before sample collection window. A relatively small proportion of samples collected from these new-borns, limited our ability to comment on model performance in these sub-groups. II) We do have a smaller sample size as compared to developed country studies [6,14,15], although this is the largest sample available from LMIC settings for metabolic profile. III) Algorithm-derived GAs tended to be overestimated in preterm infants and underestimated in post-term infants. Introducing a calibration slope adjustment [40] to model predictions may have improved overall model performance in this external cohort; however, that was not undertaken.

Our findings provide some evidence that gestational dating models developed using metabolic data derived from a North American cohort perform well in low-resource populations. These estimates are an improvement over the currently used postnatal GA estimation methods that produce estimates varying in accuracy from 2 to 4 weeks GA [4,10,12,13,41]. Difference in GA at birth of a week has a significant impact on neonatal morbidity, mortality, and long-term outcomes [42,43].

While considering implementation of metabolic gestational dating approaches for robust population-level estimates (as a replacement for current inaccurate methods), some challenges and hence opportunities need consideration. Heel prick samples for new-born screening are typically collected at least 24 hours after birth to accommodate postpartum fluctuations in metabolite levels. This introduces a bias due to early deaths selectively occurring in preterm births, further in LMIC settings most mother-infant pairs do not stay in hospital beyond 24 hours after delivery [44]. In populations where such samples are routinely collected by the health system, scaleup as is makes sense. However, in most LMIC, new-born screening is not a standard practice and will entail challenges in sample collection and processing for metabolic screening. Therefore, rethinking and research investigating modifications to this approach are needed. These include development of cord-blood-specific models restricted to metabolites less susceptible to fluctuations in the postnatal environment, establishing a profile of fewer selected metabolites that are measurable in less sophisticated equipment.

One limitation in such endeavours has been lack of characterised large cohorts in LMIC to build revised algorithms and models. Need for use of additional local reference data for algorithm development is also apparent from lack of accuracy of current models in SGA new-borns and observed differences between Asia and Africa. Newer machine learning approaches and introduction of advanced analytical methods may aid in improving accuracy of estimation.

Currently reported GA model approaches (3 sites – Iowa, Ontario, California) are restricted to traditionally obtained new-born screening metabolites. While rethinking and investigating low-tech variations suitable to LMIC settings, also to consider are, newer high throughput trans proteome/metabolome platforms which are now becoming affordable (ie, Seers Nano peptide technology [45], Precision biomarker laboratory [46], Sapient Bioanalytics [47]. An untargeted metabolomic approach may improve our ability to estimate GA postnatally while also identifying infants at risk of a variety of conditions. Use of a broader spectrum of metabolites may also help select a restrictive model for cord blood. Metabolic GA dating at present aims to provide population-based estimates of preterm birth and SGA burden, it is conceivable with introduction of advanced analytical methods and machine learning approaches could also guide care for high-risk newborns.

## CONCLUSION

Algorithms and regression coefficients generated in Iowa were externally valid in South Asia and Sub Saharan Africa. Metabolic gestational age dating approaches offer a novel means for providing accurate population-level gestational age estimates in LMIC setting and help implementing preterm birth surveillance initiatives. A global guideline for the level of acceptable accuracy of metabolic algorithms is needed. Further research should focus on use of advanced analytic methods providing broader than conventional metabolic screen analytes. Coupled with machine learning, this could enable evaluating region-specific, broad untargeted or more specific feasible metabolite approaches. Derivation and optimization of cord blood metabolic profiles models predicting gestational age accurately being an obvious focus of such efforts.
